# Acceptance Commitment Therapy Intervention for Custodial Grandfamilies

**DOI:** 10.1093/geroni/igaa057.3403

**Published:** 2020-12-16

**Authors:** Manuela E Faulhaber, Amie Zarling, Jeongeun Lee

**Affiliations:** Iowa State University, Ames, Iowa, United States

## Abstract

Millions of American children under the age of 18 are being cared for by their grandparents and without the presence of the biological parents. The number of custodial grandfamilies has significantly increased over the last five years. Recent studies have shown that custodial grandparents (CPGs) are often facing specific challenges in life, such as lower emotional well-being, higher parenting burden and stress related to this unique situation. Despite these findings, few interventions take a strengths based approach to improve their mental health and resilience. We describe our efforts to address these issues by proposing intervention anchored in the Acceptance and Commitment Therapy (ACT), emphasizing the importance of acceptance of challenging circumstances outside of one’s control and promoting resilience among participants. The program consists of a web based ACT program with online coaching meetings, six common core sessions and six separate sessions for each age group over a time period of six months. This program is unique in the sense that it utilizes both individual and group session techniques to facilitate the learning process. Main active ingredients of this program are to promote effective coping strategies, to reduce parenting stress among grandparents and to increase life skills (i.e., decision-making, proactivity) among grandchildren. We are hypothesizing that participating in the ACT program will help CGPs to improve self-efficacy, emotional well-being, higher self-confidence, social competence, lower depressive symptoms, and parenting distress, thereby leading to positive outcomes such as improved mental health and higher resilience.

